# Management of Rituximab-Associated Hypersensitivity Reactions with Successfully Applied Desensitization Protocols: A Clinical Experience of 46 Infusions in 11 Patients

**DOI:** 10.3390/jcm15114164

**Published:** 2026-05-28

**Authors:** Ömer Candar, Vildan Özkocaman, Raziye Tülümen Öztürk, Tuba Ersal, Esra Gülderen, Cumali Yalçın, Sinem Çubukçu, Tuba Güllü Koca, Fazıl Çağrı Hunutlu, Şeyma Yavuz, Dane Ediger, Fahir Özkalemkaş

**Affiliations:** 1 Division of Hematology, Department of Internal Medicine, Faculty of Medicine, Bursa Uludağ University, 16059 Bursa, Turkey; vildanoz@uludag.edu.tr (V.Ö.); tubaersal@uludag.edu.tr (T.E.); doktorcumali@hotmail.com (C.Y.);; 2 Department of Hematology, Faculty of Medicine, Bursa Uludag University, 16059 Bursa, Turkey; 3 Division of Allergy and Immunology, Department of Chest Diseases, Faculty of Medicine, Bursa Uludağ University, 16059 Bursa, Turkey; rtulumenozturk@uludag.edu.tr (R.T.Ö.); ediger@uludag.edu.tr (D.E.); 4 Department of Internal Medicine, Faculty of Medicine, Bursa Uludağ University, 16059 Bursa, Turkey

**Keywords:** rituximab, allergy, desensitization, lymphoma, leukemia

## Abstract

**Objective:** This study aimed to evaluate patients who developed hypersensitivity reactions (HSRs) during rituximab treatment and report the outcomes of desensitization protocols implemented to allow treatment continuation. **Methods:** We retrospectively reviewed the institutional data of 76 patients who received rituximab therapy at the Adult Hematology Department between January 2022 and September 2023. Among these, 11 patients who experienced immediate hypersensitivity reactions during infusion were analyzed. The overall frequency of rituximab-associated HSRs was 14.47% (11 out of 76 patients). Demographic data, underlying diseases, timing and type of HSRs, and details of the desensitization protocols were recorded. **Results:** The overall frequency of rituximab-associated HSRs was 14.47% (11 out of 76 patients). Among the 11 patients, eight were male and three were female, with a median age of 56 years (range: 19–72). Eight patients had CD20-positive non-Hodgkin lymphoma (NHL) and three had acute B-lymphoblastic leukemia (B-ALL). HSRs occurred during the first rituximab exposure in nine patients, at the fourth dose in one patient, and at the eighth dose in another. Symptoms included widespread rash, pruritus, flushing, chills, shivering, dyspnea, dysphagia, back pain, dizziness, syncope, and throat discomfort. All the patients were consulted by the Allergy and Immunology Clinic. Based on prick and intradermal test (IDT) results and the planned rituximab dose, desensitization protocols consisting of a three-dilution/12-step and a four-dilution/16-step regimen were prepared. Overall, 46 desensitization procedures were successfully completed in 11 patients. Notably, no severe anaphylactic events or treatment discontinuations due to drug toxicity occurred during the implementation of the protocols. **Conclusions:** Although the number of patients was limited, our findings indicate that in patients with hematologic malignancies receiving rituximab who develop early HSRs, desensitization represents a safe and effective strategy before considering treatment modification. These results support that, in appropriately selected patients, desensitization protocols are an important approach to continue therapy without interruption while minimizing adverse reactions.

## 1. Introduction

Monoclonal antibodies (mAbs) have become essential for therapies targeting neoplastic conditions and for chronic inflammatory and autoimmune diseases [1,2,3,4,5,6]. Rituximab is a chimeric IgG monoclonal antibody that targets the CD20 antigen expressed on normal and malignant B-cells [7]. It was initially approved as an anti-neoplastic agent [8]. Subsequently, it has emerged as a promising therapeutic option for chronic granulomatous and inflammatory diseases as an alternative to conventional therapy [9].

Rituximab therapy can lead to adverse reactions in two main categories: immunodeficiency and hypersensitivity reactions (HSRs). The drug is generally well-tolerated and considered acceptable [10,11]; however, the increasing use of rituximab has been associated with HSRs [4,5,12]. Comprehensive reviews focusing on rituximab-associated HSRs are quite limited.

HSRs can be type 1 immunoglobulin E/non-immunoglobulin E (IgE/Non-IgE)-mediated (pruritus, urticaria, dyspnea, hypotension, and anaphylaxis); cytokine-mediated (fever, chills, rigors, nausea, pain, headache); or of a mixed type [13,14]. In type 1 IgE/non-IgE reactions, the degranulation of mast cells and basophils results in a massive release of histamine, leukotrienes, and prostaglandins, which triggers clinical symptoms. Cytokine-mediated clinical symptoms, on the other hand, have been associated with elevated levels of Tumor Necrosis Factor-alpha (TNF-α) and Interleukin-6 (IL-6) [4,9,15].

If an HSR occurs within 48 h of infusion, it is termed an early-type HSR; if it occurs after 48 h, it is considered a delayed-type HSR. This high incidence of infusion reactions, reported to occur in 25% to 78% of cases [9], makes rituximab one of the most prominent culprits among biologic therapies. In current oncology and hematology practice, immediate HSRs are frequently encountered with platinum-based chemotherapeutic agents (such as carboplatin and oxaliplatin) and taxanes (such as paclitaxel). Among these agents, rituximab stands out as one of the most prominent culprits. The vulnerability to rituximab-induced reactions is primarily attributed to its chimeric structure, which significantly increases its immunogenic potential compared to fully humanized monoclonal antibodies. Depending on the underlying immunological mechanism—whether cytokine release or IgE-mediated anaphylaxis—these reactions can manifest during the patient’s very first exposure to the drug or develop during subsequent cycles due to progressive sensitization [16]. When standard infusions fail due to such presentation, implementing an effective and resource-optimized rapid drug desensitization (RDD) protocol becomes a clinical necessity to ensure the safe continuation of this essential therapy.

Skin tests are critical both for understanding the nature of the original hypersensitivity reaction and for predicting the risk of a reaction during the desensitization (DS) process. Skin test screening performed before the initial administration of rituximab and a planned desensitization protocol in appropriately selected patients can reduce both the incidence and clinical severity of immediate hypersensitivity reactions. Specifically, the result of an IDT performed before the first infusion can be a clinically valuable predictor for the development of rituximab-associated early HSRs [17].

Desensitization is a procedure that modifies the immune response to induce a temporary state of clinical tolerance to an allergenic drug by gradually increasing its doses at specific time intervals. It is generally preferred by experienced centers in cases where alternative treatment options are unavailable or pose a significant risk to the patient’s health status. It is effective in patients with early-onset hypersensitivity symptoms, including anaphylaxis, that are type 1 IgE/non-IgE-mediated or cytokine-mediated. Drug desensitization is not performed for delayed drug reactions such as Stevens–Johnson syndrome, toxic epidermal necrolysis, serum sickness, or hemolytic anemia [18,19]. Castells et al. (2008) first reported successful outcomes with desensitization in three cases of Rituximab HSRs. In that study, 12-step protocols with a three-dilution solution were used [16]. For patients who develop HSRs despite 12-step protocols, 16-step protocols with a four-dilution solution can also be used [18].

In our study, we have shown that three-dilution/12-step and four-dilution/16-step desensitization protocols can be safely and effectively administered in patients with hematologic malignancies who developed hypersensitivity during rituximab therapy. During the 46 procedures performed in 11 patients, all the patients were able to complete the therapy, and no serious complications were observed. These findings support that in situations where rituximab is essential, alternative potent therapies are limited, and cost-effectiveness is a significant consideration, desensitization is a strategy that can be safely applied without treatment modification or discontinuation. Similarly, larger case series in the literature have reported that rapid desensitization protocols for rituximab and other biologic agents can be implemented with high success rates and a low frequency of severe adverse reactions. Thus, our findings as a single-center experience demonstrate outcomes consistent with the literature, both in terms of clinical safety and treatment continuation.

## 2. Materials and Methods

We retrospectively reviewed the institutional data of 76 patients who received rituximab therapy at the Adult Hematology Department between January 2022 and September 2023. Among these, 11 patients who experienced immediate hypersensitivity reactions during infusion were analyzed. The overall frequency of rituximab-associated HSRs was 14.47% (11 out of 76 patients). Demographic data, underlying diseases, timing and type of HSRs, and details of the desensitization protocols were recorded. Patients under eighteen years of age and those who exhibited delayed-type hypersensitivity reactions were excluded from the study. The ECOG performance status at diagnosis, history of atopy, age at diagnosis, type and grade of the HSR, skin tests performed, premedication therapies received, dose of rituximab received, the administered 12- and 16-step desensitization protocols, and treatment outcomes were evaluated for all patients.

The drug desensitization process was performed using two distinct mathematical models based on the patient’s clinical tolerance. For the standard cohort, a 3-dilution, 12-step protocol was implemented as the primary therapeutic approach. In this model, three separate drug concentrations (Solutions 1, 2, and 3) were prepared, with each subsequent solution being 10-fold more concentrated than the previous one (1:100, 1:10, and 1:1, respectively). The infusion rate was doubled every 15 min across 12 consecutive steps, allowing a gradual and controlled exposure to the target dose.

For cases demonstrating resistant hypersensitivity reactions where the standard protocol failed, the strategy was modified into a 4-dilution, 16-step protocol. To clear the clinical confusion between these two distinct pathways, the 16-step protocol introduced an extra, highly diluted baseline solution (Solution 1:1000) administered through 4 additional initial steps. This architectural expansion ensured a much slower antigen challenge for highly sensitized patients. Due to the high density of steps and data fields required to visualize these multi-dilution rates, the technical specifications for both protocols have been detailed in Supplementary Tables S2 and S3, which are formatted in a landscape configuration for optimal structural clarity.

### 2.1. Ethical Approval

Ethics approval was obtained from the Uludağ University Faculty of Medicine Clinical Research Ethics Committee (Approval date: 19 September 2023, Approval number: 2023-17/55). All aspects of the study, including periodic clinical and laboratory checkups, were performed in accordance with the principles of the Declaration of Helsinki (64th, 2013). Written informed consent was obtained from all the participants prior to inclusion in the study.

### 2.2. Declaration of Patient Consent

The authors certify that they have obtained all the appropriate patient consent forms. In the form, the patient(s) has/have given his/her/their consent for his/her/their images and other clinical information to be reported in the journal. The patients understand that their names and initials will not be published and due efforts will be made to conceal their identity, but anonymity cannot be guaranteed.

## 3. Results

Of the 76 patients treated with rituximab at our institution between January 2022 and September 2023, 11 patients developed rituximab-induced hypersensitivity reactions (HSRs), demonstrating an overall institutional HSR frequency of 14.47%. The median age of the 11 patients included in the study was 56 years (range: 19–72). Among the patients, eight were male and three were female. Three patients had CD20-positive acute B-lymphoblastic leukemia, while eight patients had B-cell non-Hodgkin lymphoma (Table 1). None of the patients had a history of atopy. We determined that HSRs developed during the first exposure to rituximab in nine patients, and at subsequent doses in the other two patients (one after the fourth dose and the other after the eighth dose of rituximab therapy). The HSRs were generally between Grades 1 and 3, and no Grade 4 or 5 reactions were observed in any of our patients. These reactions typically occurred 30 min after the rituximab infusion began. HSRs were graded according to the National Cancer Institute’s Common Terminology Criteria for Adverse Events, version 5.0 (NCI-CTCAE v5.0) (Supplementary Table S1). Notably, no isolated or self-limiting Grade 1 hypersensitivity reactions (HSRs) were recorded in our cohort. In the patients who initially presented with mild, early symptoms, these signs rapidly overlapped and co-existed with Grade 2 clinical manifestations. This immediate progression necessitated prompt, protocol-driven breakthrough premedication and bedside intervention directly on the clinical ward to halt further systemic escalation. The characteristics of the HSRs are shown in Table 2. While the majority of the patients developed HSRs during their initial rituximab exposure (Cycle 1), subsequent-cycle reactions were observed in two patients, indicating drug sensitization. Specifically, Patient 3 developed an HSR during the fourth cycle (approximately day 63 of overall therapy), and Patient 4 developed an HSR during the eighth cycle (approximately day 147 of overall therapy). For both patients, rituximab administrations were scheduled at standard 21-day intervals based on their primary treatment protocols. The most frequently observed symptom was dyspnea.

All the patients were consulted by the Allergy and Immunology Clinic. Based on the results of prick and intradermal tests (IDT) with rituximab and the planned dose for the patients, a desensitization protocol consisting of a three-dilution solution and 12 steps was initially prepared (Supplementary Table S2). In seven of our patients who developed HSRs after the first infusion, prick and IDT performed 2 weeks later were positive, while no tests were performed on four patients.

Thirty minutes prior to the desensitization procedure, all the patients received premedication with 40 mg of methylprednisolone and one ampoule of pheniramine hydrogen maleate. With the exception of one patient, no HSRs recurred at any step of the protocol in patients undergoing the 12-step desensitization. In one patient, recurrent dyspnea and widespread pruritus occurred at the second step of the first solution. The infusion was paused, and the patient was re-administered 40 mg of methylprednisolone and one ampoule of pheniramine hydrogen maleate. Thirty minutes later, the infusion was resumed. The HSRs recurred in the same manner within 5 min of the infusion. The patient was again consulted by the Allergy and Immunology Clinic. A desensitization protocol consisting of a four-dilution solution and 16 steps was administered to the patient (Supplementary Table S3). The therapy was successfully completed, with no further HSRs developing during this protocol.

## 4. Discussion and Conclusions

Hypersensitivity reactions (HSRs) following rituximab therapy most often occur during the first exposure to the drug. The implementation of a desensitization protocol allows patients’ therapy to continue without requiring a switch to a second-line agent that may have lower efficacy and success.

In the desensitization study by Castells et al. (2008), successful outcomes were reported with multiple chemotherapies in a total of 98 patients and 413 procedures (carboplatin, paclitaxel, cisplatin, oxaliplatin, liposomal doxorubicin, doxorubicin, and rituximab). Additionally, in this study, intraperitoneal desensitization with paclitaxel and cisplatin was administered to patients with ovarian cancer [16]. In a study by Brennan P. J. et al. (2009), 105 desensitization procedures with monoclonal antibodies (rituximab, infliximab, and trastuzumab) were successfully administered in 23 patients [12]. In both studies, a three-dilution, 12-step protocol was administered. Although mild to moderate HSRs developed during the desensitization procedures in these studies, the protocol was continued after symptomatic treatment and successfully completed. In our study, the three-dilution, 12-step protocol for rituximab therapy was successfully administered. However, because one of our patients developed a resistant HSR, a four-dilution, 16-step protocol was administered and successfully completed.

In the literature, the reported incidence of rituximab-associated adverse infusion events spans a wide spectrum. Recently, a retrospective study demonstrated a remarkably high infusion-related reaction (IRR) rate of 47.2% (119/252) among patients with NHL [20]. Notably, those findings emphasized that the incidence was significantly more pronounced in specific histological subtypes, reaching up to 59.0% in follicular lymphoma cases, driven mainly by a high pre-treatment lymphocyte count and elevated cytokine/chemokine levels [20]. In comparison, our institutional cohort presented an overall hypersensitivity reaction (HSR) frequency of 14.47% (11/76). While our incidence remains relatively lower, the significant clinical challenge highlighted in the literature underscores the absolute necessity of robust, resource-optimized risk management solutions. When standard infusions must be permanently discontinued due to moderate-to-severe hypersensitivity presentations, implementing RDD protocols—as successfully applied in 46 infusions within our series—serves as an indispensable and highly safe clinical tool. While no baseline severe anaphylactic events (Grades 4/5) were recorded in our cohort initially, the implementation of our desensitization strategy successfully prevented any further systemic escalation or breakthrough reactions during subsequent cycles, ensuring the safe, uninterrupted, and complete delivery of this essential chimeric monoclonal antibody entirely on the standard clinical ward.

In a three-case study by Yang B. C. et al. (2019) on patients who developed HSRs with rituximab, it was reported that one patient developed severe reactions such as life-threatening angioedema and anaphylaxis. The patient’s therapy was immediately discontinued, and intramuscular epinephrine, intravenous glucocorticoid, and crystalloid were administered. They published that three weeks later, after the patient was consulted by the immunology department and an intradermal rituximab test yielded a positive result, they successfully administered a four-dilution, 16-step rituximab desensitization procedure. In the same study, all the patients were administered cetirizine 10 mg p.o. and famotidine as H1 and H2 blockers for premedication [13]. In our study, severe reactions such as anaphylaxis and angioedema did not occur, and we administered 40 mg of methylprednisolone and one ampoule of pheniramine maleate as premedication 30 min prior to rituximab therapy.

Öztürk E. et al. (2017) reported the successful administration of desensitization therapy in three patients diagnosed with non-Hodgkin lymphoma who developed HSRs following rituximab therapy [18]. The three patients who developed HSRs had no known history of atopy. HSRs developed during the first exposure to rituximab therapy in two of the patients, while in the other patient, it occurred during the second exposure. The median age of the patients was 39 years (range: 37–60), and all three patients were male. One patient developed a severe anaphylactic reaction, including hypotension and incontinence, while the other two patients developed moderate HSRs. The patient who developed the severe HSRs was administered the four-dilution, 16-step desensitization protocol under intensive care unit conditions, while the other patients received the three-dilution, 12-step desensitization protocol on the clinical ward. In one patient, a mild delayed erythema, which was controlled with antihistamines, was observed on the 2nd day of desensitization, and this occurred after each desensitization procedure. The therapy for the three patients who developed HSRs was not continued on the same day but was administered via desensitization 48 h later. The three patients received a total of 13 desensitization procedures and were administered premedication with H1 and H2 blockers, montelukast sodium, and a systemic steroid.

In a study by Johnson T. Wong et al. (2017), in which 170 desensitization procedures were administered to 25 patients receiving rituximab, three patients developed delayed-type HSRs. One patient experienced serum sickness, and a second patient experienced severe arthralgia on the 7th day of therapy. The third patient developed delayed-onset urticaria and angioedema on the 5th day of therapy. The therapies for these three patients were successfully completed with corticosteroids administered as premedication [9]. In our study, a total of 46 desensitization procedures were administered to 11 patients. Eight of the patients had NHL, and three had CD20-positive B-ALL. Eight patients were male, and three were female, with a median age of 56 years (range: 19–72). As no severe HSRs, such as anaphylaxis, developed, initially, the standard three-dilution, 12-step desensitization protocol was initiated for all the patients on the clinical ward. Except for one patient who required a transition to the four-dilution, 16-step protocol due to a resistant reaction, all the procedures were successfully completed using the standard regimen entirely on the regular floor. None of the patients had a history of atopy, and no patient developed a delayed-onset HSR. In all the patients, therapy was not continued on the same day after the HSRs developed; instead, the therapy was completed via desensitization administered at least 2 weeks later.

While larger cohorts and multicenter studies established the fundamental feasibility of rituximab desensitization, our single-center experience communicates distinct practical improvements in clinical workflow and resource optimization. In our cohort, which uniquely features a detailed description of CD20-positive B-ALL patients alongside NHL cases, we demonstrated that standard and even resistant hypersensitivity cases can be safely managed entirely within a standard clinical ward. Unlike some reported protocols in the literature that required intensive care unit (ICU) admission for 16-step regimens due to severe reactions, our transition from a failed 12-step protocol to a successful four-dilution/16-step protocol was accomplished safely on the clinical floor without any severe complications. This protocol flexibility minimizes ICU dependency, significantly reduces hospital resource utilization, and provides a highly cost-effective model for sustaining essential first-line monoclonal antibody therapies.

In a single-center study by Görgülü B. et al. (2019) involving a series of 24 patients, they emphasized that HSRs developed in 20 of the patients during their first exposure to rituximab, while in the other four patients, reactions occurred after subsequent administrations. They performed a total of 141 desensitization procedures. HSRs, such as dyspnea, occurred in 22 of the twenty-four patients. All the patients except for two completed the target dose. Severe anaphylaxis, including profound hypotension and widespread urticaria, occurred in two patients. These two patients were administered half the target dose with a 16-step therapy [21]. In our study, dyspnea was also present in seven of the 11 patients. No severe anaphylaxis occurred in any patient. Ten of our patients completed the target dose with the 12-step desensitization protocol, while one patient completed it with the 16-step protocol.

To better contextualize our outcomes within the current medical literature, a detailed structural and operational comparison between our clinical experience and previously published rapid desensitization protocols (including Castells et al., 2008 [16]; Brennan et al., 2009 [12]; Yang et al., 2019 [13]; Öztürk et al., 2017 [18]; Görgülü et al., 2019 [21]; and Unutmaz Erkaya et al., 2024 [22])is systematically presented in Table 3. Unlike several protocols in the literature that mandate ICU admission for advanced or modified steps, our single-center experience highlights a proactive and dynamic ward-management model. The absence of isolated Grade 1 reactions in our study is reflective of our immediate clinical response; as soon as early, transient symptoms emerged, they rapidly evolved alongside Grade 2 signs, triggering immediate abortive breakthrough premedications. This prompt bedside intervention effectively controlled reaction kinetics, confirming that even standard-protocol-resistant cases can be safely transitioned to a 16-step pathway and successfully managed entirely within a standard clinical ward without compromising patient safety or resource optimization. This comparative overview explicitly highlights the unique resource-management advantages and high target-dose completion rates achieved by our single-center protocol design.

In a study by Unutmaz Erkaya D. G. et al. (2024) with 45 patients and nine different biologic agents, the most severe HSRs that developed were Grade 3, and they successfully administered a 12-step desensitization protocol. They found no significant difference between positive or negative IDT results and the development of HSRs [22]. In a study by Li L. et al. (2021) with 19 patients, the positive predictive value of IDTs performed before the initial administration of rituximab was found to be 100%, while the negative predictive value was 84.6%, and the HSRs that developed were at least Grade 3 [17]. In our study, the most severe HSRs observed were also Grade 3; however, IDT was performed on these seven patients 2 weeks after the first infusion.

In conclusion, our single-center experience and the systematic literature comparison presented in Table 3 demonstrate that, particularly in situations where rituximab is essential, alternative potent therapies are limited, or cost-effectiveness is a key clinical driver, treatment discontinuation can be successfully avoided. Beyond validating the absolute safety and feasibility of rapid drug desensitization, our outcomes uniquely contribute a highly practical, flexible, and resource-optimized model by showing that even standard-protocol-resistant hypersensitivity cases can be successfully managed and titrated to a 100% target dose entirely within a standard clinical ward, providing a viable blueprint for overcoming challenging biologic reactions without relying on intensive care interventions.

## Figures and Tables

**Table 1:** Frequency of patient diagnoses.

Diagnosis	Frequency	Percentage (%)
DLBCL	4	36.4
B-ALL	3	27.3
FL	2	18.2
HCL	1	9.1
MZL	1	9.1
Total	11	100.0

DLBCL: diffuse large B-cell lymphoma; B-ALL: B-cell acute lymphoblastic leukemia; FL: follicular lymphoma; HCL: hairy cell leukemia; MZL: marginal zone lymphoma.

**Table 2:** Characteristics of HSRs in patients following rituximab therapy and the number of desensitizations.

Patient	Symptom	Dose of HSRs Onset	Number of Desensitizations
1	Dyspnea, dizziness, chills, shivering	1	5
2	Throat discomfort, dyspnea, throat tightness	1	3
3	Syncope, dyspnea, chest pain	4	4
4	Flushing, back pain	8	4
5	Pruritus, dysphagia	1	7
6	Pruritus, fever, widespread rash	1	4
7	Dyspnea, inability to swallow, fever	1	5
8	Dyspnea	1	3
9	Chest pain, palpitations	1	3
10	Dyspnea, throat tightness	1	5
11	Dyspnea, widespread pruritus, fever	1	3

Frequency of symptoms during initial hypersensitivity reactions in the 11 patients: dyspnea (*n* = 7), throat tightness (*n* = 4), rash/pruritus (*n* = 4), fever (*n* = 3), dysphagia (*n* = 2), dizziness (*n* = 1), chills/shivering (*n* = 1), chest pain (*n* = 1), and palpitations (*n* = 1).

**Table 3:** Comparison of the present rituximab desensitization protocol with previous literature protocols.

Study (Reference)	Patient Cohort/ Diagnoses	Protocol Design (Steps and Dilutions)	Management Setting	Key Clinical/Operational Outcome and Novelty
Castells et al. [16]	98 patients (Mixed chemo and mAbs)	Standard 12-step (3 dilutions)	Specialized Allergy Unit/ICU	First described the foundational feasibility of the 12-step multi-dilution model.
Brennan et al. [12]	23 patients (Rituximab, Infliximab, Trastuzumab)	Standard 12-step (3 dilutions)	Specialized Outpatient/Inpatient	Confirmed safety of 12-step rapid desensitization for monoclonal antibodies.
Yang et al. [13]	3 patients (Rituximab)	16-step (4 dilutions) for severe cases	Specialized Immunology Center	Utilized H1/H2 blockers (cetirizine and famotidine) for premedication for severe reactions.
Öztürk et al. [18]	3 patients (NHL)	12-step (3 dilutions) and 16-step (4 dilutions)	Intensive Care Unit (ICU) for 16-step	Completed 16-step protocol strictly under ICU conditions due to severe anaphylaxis.
Görgülü et al. [21]	24 patients (Rituximab)	12-step (3 dilutions) and 16-step (4 dilutions)	Standard Ward/Clinical Floor	Achieved high success, but 2 patients with severe anaphylaxis could only receive half the target dose.
Unutmaz Erkaya et al. [22]	45 patients (9 different biologic agents)	Standard 12-step protocol	Multicenter/Specialized Centers	Evaluated multiple biologics, reported up to Grade 3 reactions, and found no significant correlation between skin test positivity and HSR development.
Present Study (Candar et al.)	11 patients (NHL and CD20+ B-ALL)	Standard 12-step (3 dilutions) and Modified 16-step (4 dilutions)	Entirely on the Standard Clinical Ward (No ICU required)	Demonstrated that even resistant cases failing the 12-step protocol can be safely transitioned to a 16-step protocol entirely on the regular floor, achieving 100% target dose completion with optimized hospital resources.

## Data Availability

The datasets generated and/or analyzed during the current study are not publicly available due to patient privacy and ethical restrictions. However, they are available from the corresponding author upon reasonable request.

## Supplementary Materials

The following supporting information can be downloaded at https://www.mdpi.com/article/10.3390/jcm15114164/s1. Table S1: The National Cancer Institute’s Common Terminology Criteria for Adverse Events, version 5.0; Table S2: Standard 3-dilution, 12-step rituximab desensitization protocol: Preparation solution and administration schedule (for 750mg of rituximab); Table S3: Modified 4-dilution, 16-step rituximab desensitization protocol for resistant cases: Preparation solution and administration schedule (for 672 mg of rituximab).
