# Supplementary material for: Management of Rituximab-Associated Hypersensitivity Reactions with Successfully Applied Desensitization Protocols: A Clinical Experience of 46 Infusions in 11 Patients
Source: J Clin Med. 2026 May 28;15(11):4164. doi: 10.3390/jcm15114164 (PMC13257869; doi:10.3390/jcm15114164)
Supplement: Supplementary file 1 [file jcm-15-04164-s001.zip › Supplementary Table S1.pdf]

Supplementary Table S1. The National Cancer Institute's Common Terminology Criteria for Adverse Events, version 5.0.

Grade	Definition	Clinical Findings
**Grade 1 (Mild)**	Minimal reaction, no intervention required; tolerated without treatment interruption	Mild rash, pruritus, mild flushing, mild chills, restlessness
**Grade 2 (Moderate)**	Requires minimal medical intervention or medication; temporary interruption of infusion may be necessary	Widespread rash, moderate dyspnea, wheezing, tachycardia without hypotension, fever, chills, shivering
**Grade 3 (Severe)**	Pronounced symptoms, not life-threatening but requiring urgent intervention	Bronchospasm, hypoxia, pronounced dyspnea, angioedema, hypotension, syncope
**Grade 4 (Life-threatening)**	Requires urgent intervention; severe hemodynamic or respiratory collapse	Anaphylaxis, cardiopulmonary arrest, severe hypoxia or hypotension
**Grade 5 (Death)**	Fatal reaction	Death resulting from cardiac arrest or multi-organ failure
