# Supplementary material for: Management of Rituximab-Associated Hypersensitivity Reactions with Successfully Applied Desensitization Protocols: A Clinical Experience of 46 Infusions in 11 Patients
Source: J Clin Med. 2026 May 28;15(11):4164. doi: 10.3390/jcm15114164 (PMC13257869; doi:10.3390/jcm15114164)
Supplement: Supplementary file 1 [file jcm-15-04164-s001.zip › Supplementary Table S2.pdf]

Supplementary Table S2. Standard 3-dilution, 12-step rituximab desensitization protocol: Preparation solution and administration schedule (for 750mg of rituximab).

	Volume	Concentration	Total dose (mg)	Volume infused (ml)
Solution A	250 ml	0,0300	7.5	9.38
Solution B	250 ml	0.3000	75	18.75
Solution C	250 ml	2.9700	742.5	250

Step	Solution	Rate (ml/h)	Duration (min)	Dose administered	Cumulative dose
1	A	2.5	15	0.0188	0.019
2	A	5	15	0.0375	0.056
3	A	10	15	0.0750	0.131
4	A	20	15	0.1500	0.281
5	B	5	15	0.3750	0.656
6	B	10	15	0.7500	1.406
7	B	20	15	1.5000	2.906
8	B	40	15	3.0000	5.906
9	C	10	15	7.4250	13.331
10	C	20	15	14.8500	28.181
11	C	40	15	29.7000	57.881
12	C	75	186	692.1188	750.000

The total administration time was 351 minutes (5.85 hours).
