# Supplementary material for: Management of Rituximab-Associated Hypersensitivity Reactions with Successfully Applied Desensitization Protocols: A Clinical Experience of 46 Infusions in 11 Patients
Source: J Clin Med. 2026 May 28;15(11):4164. doi: 10.3390/jcm15114164 (PMC13257869; doi:10.3390/jcm15114164)
Supplement: Supplementary file 1 [file jcm-15-04164-s001.zip › Supplementary Table S3.pdf]

Supplementary Table S3. Modified 4-dilution, 16-step rituximab desensitization protocol for resistant cases: Preparation solution and administration schedule (for 672 mg of rituximab).

		Volume	Concentration		Total dose (mg)	Volume infused (ml)
Solution A		250 ml	0.0027		0.672	9.38
Solution B		250 ml	0.0269		6.72	9.38
Solution C		250 ml	0.2688		67.2	18.75
Solution D		250 ml	2.6853		671.328	230
**Step**	**Solution**		**Rate (ml/h)**	**Duration (min)**	**Dose administered**	**Cumulative dose**
1	A		2.5	15	0.0017	0.0017
2	A		5	15	0.0034	0.0050
3	A		10	15	0.0067	0.0118
4	A		20	15	0.0134	0.0252
5	B		2.5	15	0.0168	0.042
6	B		5	15	0.0336	0.0756
7	B		10	15	0.0672	0.1428
8	B		20	15	0.1344	0.2772
9	C		5	15	0.3360	0.6132
10	C		10	15	0.6720	1.2852
11	C		20	15	1.3440	2.6292
12	C		40	15	2.6880	5.3172
13	D		10	15	6.7133	12.030
14	D		20	15	13.4266	25.457
15	D		30	15	26.8531	52.3101
16	D		40	345	619.689	672.000

The total administration time was 570 minutes (9.5 hours).
